# Microbiological and Infection-Source Predictors of Mortality in Severe Sepsis Patients Undergoing Polymyxin B Hemoperfusion: A Seven-Year Real-World Cohort Study

**DOI:** 10.3390/life16010121

**Published:** 2026-01-13

**Authors:** Wei-Hung Chang, Li-Kuo Kuo, Kuan-Pen Yu, Ting-Yu Hu

**Affiliations:** 1 Department of Critical Care Medicine, MacKay Memorial Hospital, Taipei 10449, Taiwan; peacejaycool@gmail.com (W.-H.C.); dennispipi77@gmail.com (K.-P.Y.); 2 Department of Medicine, Mackay Medical College, New Taipei City 25245, Taiwan

**Keywords:** polymyxin B hemoperfusion, sepsis, septic shock, infection source, multidrug resistance, intensive care unit, vasoactive–inotropic score

## Abstract

Background: The microbiological landscape and infection-source profiles of severe sepsis in Asian ICUs differ markedly from Western cohorts and may influence the effectiveness and prognosis of adjunctive therapies such as polymyxin B hemoperfusion (PMX-HP). However, real-world data on how pathogen categories, multidrug resistance (MDR), and infection sources affect outcomes in PMX-HP-treated patients are lacking. Methods: We conducted a retrospective cohort study in a tertiary medical ICU in Taiwan, including adult patients with severe sepsis or septic shock who received PMX-HP between 2013 and 2019. Microbiological data, infection sources, MDR profiles, organ support requirements, vasoactive–inotropic score (VIS), and mortality outcomes were retrieved from electronic records. Pathogen groups (Gram-negative, Gram-positive, fungal, no-growth), MDR status, and infection sources were analyzed for associations with 28-day, ICU, and hospital mortality. Results: Among 64 patients (mean age 66.1 years; 67.2% male), Gram-negative pathogens predominated (70.3%), with *Escherichia coli* (31.3%) and *Klebsiella pneumoniae* (21.9%) being the most frequently identified organisms. MDR organisms were isolated in 26.6% of patients. The most common infection sources were pneumonia (29.7%), intra-abdominal infection (18.8%), and urinary tract infection (17.2%). Gram-negative infections were associated with higher CRRT utilization (71.9% vs. 47.1%, *p* = 0.04) and higher VIS at 24 h. MDR status was significantly associated with early CRRT requirement (64.7% vs. 38.6%, *p* = 0.048), but not with 28-day mortality (52.9% vs. 43.2%, *p* = 0.42). No infection source was independently associated with mortality after adjustment for APACHE II, CRRT, and VIS. Instead, greater organ failure severity—particularly renal failure requiring CRRT—was strongly associated with mortality in this cohort. Conclusions: In PMX-HP-treated severe sepsis patients, Gram-negative predominance and MDR status were associated with increased organ support requirements but were not independently associated with mortality. Outcomes were primarily associated with overall illness severity rather than microbiological category. These findings highlight the importance of combining microbiological data with dynamic physiological markers for prognostic risk stratification in Asian ICUs.

## 1. Introduction

Severe sepsis and septic shock remain among the leading causes of morbidity and mortality in intensive care units (ICUs) worldwide. The Global Burden of Disease Study estimated that sepsis accounted for nearly 49 million cases and 11 million deaths globally in 2017, representing almost 20% of all annual deaths [1]. Despite advances in early recognition, antimicrobial stewardship, and standardized resuscitation bundles, mortality from septic shock frequently exceeds 40% in high-resource settings [2]. These sobering statistics underscore the persistent clinical challenge of septic shock, characterized by dysregulated host responses, circulatory collapse, and multi-organ dysfunction.

In Taiwan and many Asian countries, the epidemiology of sepsis is distinct from Western regions. National audit data demonstrate that Gram-negative bacteria—particularly *Escherichia coli*, *Klebsiella pneumoniae*, and *Acinetobacter baumannii*—predominate in severe sepsis, in contrast to the more balanced Gram-positive and Gram-negative distribution in Western ICUs [3]. Gram-negative organisms trigger profound inflammatory responses through endotoxin (lipopolysaccharide, LPS), activating toll-like receptor 4 signaling and driving cytokine release, endothelial injury, vasoplegia, and metabolic dysregulation [4]. In contrast, Gram-positive pathogens exert their pathogenicity through mechanisms such as exotoxin production and superantigen activation, resulting in distinct immunological trajectories [5]. Infection source also substantially influences clinical course and mortality. Pneumonia, intra-abdominal infection, urinary tract infection, soft-tissue infection, and hepatobiliary sepsis each impart unique risks for organ failure and treatment delays, with implications for targeted management strategies [6].

Polymyxin B hemoperfusion (PMX-HP) was developed as an extracorporeal modality to selectively remove circulating endotoxin. Early clinical experience suggested improved hemodynamic stability, reduced vasopressor requirements, and possible survival benefits in abdominal septic shock [7,8]. These favorable early findings contributed to widespread adoption in Japan and increasing use in Taiwan, especially given the high prevalence of Gram-negative infections and the availability of National Health Insurance reimbursement. However, subsequent large randomized controlled trials (RCTs) failed to confirm consistent mortality benefit. The ABDOMIX trial did not show improved outcomes with PMX-HP [9], and the EUPHRATES trial—targeting patients with elevated endotoxin activity—also reported no overall mortality reduction [10]. These divergent results highlight ongoing uncertainty regarding the optimal use of PMX-HP and the importance of patient selection, timing, and microbiological context.

Microbiological and infection-source characteristics may critically influence the clinical effectiveness of PMX-HP. However, existing RCTs and meta-analyses have rarely stratified outcomes by pathogen category, multidrug-resistant (MDR) status, or infection source. Recent network meta-analyses and real-world analyses have emphasized the need to evaluate PMX-HP within defined biological and clinical phenotypes [11,12]. This represents a major knowledge gap, particularly in regions such as Taiwan where Gram-negative and MDR infections are highly prevalent and frequently associated with severe sepsis.

Key unanswered questions include the following: Do Gram-negative infections exhibit different hemodynamic responses or mortality trajectories under PMX-HP compared to non-Gram-negative infections? Does MDR status influence organ support requirements or attenuate response to hemoperfusion? Are certain infection sources—such as pneumonia or intra-abdominal infection—associated with improved or worsened outcomes despite PMX-HP? Finally, do microbiological characteristics independently predict mortality, or are outcomes predominantly driven by global severity metrics such as APACHE II score, vasoactive–inotropic score (VIS), and the need for continuous renal replacement therapy (CRRT)?

To address these gaps, we conducted a seven-year retrospective cohort study of adult patients with severe sepsis or septic shock treated with PMX-HP in a Taiwanese tertiary ICU. We aimed to characterize pathogen distribution, MDR patterns, and infection-source profiles and to evaluate their associations with mortality and organ support requirements. To our knowledge, this is the first dedicated analysis focusing on microbiological and infection-source predictors of outcome in PMX-HP-treated patients in Taiwan and one of the few such analyses in Asia. These findings may advance biologically informed patient selection, refine therapeutic expectations, and improve the precision use of PMX-HP in high-burden Gram-negative sepsis settings.

## 2. Materials and Methods

### 2.1. Study Design and Setting

This retrospective observational cohort study was conducted in the medical intensive care unit (ICU) of MacKay Memorial Hospital, a tertiary referral center in Taipei, Taiwan. The ICU is a closed 25-bed unit staffed by full-time intensivists and provides advanced organ support including mechanical ventilation, continuous renal replacement therapy (CRRT), and extracorporeal membrane oxygenation (ECMO). Polymyxin B hemoperfusion (PMX-HP) has been reimbursed by the Taiwan National Health Insurance since 2013 for refractory septic shock, allowing consistent implementation throughout the study period. The study covered a 7-year interval between 1 July 2013 and 31 December 2019.

### 2.2. Patient Population

All consecutive adult patients (≥20 years old) admitted with severe sepsis or septic shock who received at least one PMX-HP session were screened for eligibility.

#### 2.2.1. Inclusion Criteria

1. Age ≥ 20 years.
2. Diagnosis of severe sepsis or septic shock, based on contemporaneous Surviving Sepsis Campaign definitions:
  ○ Severe sepsis (pre–2016): sepsis with ≥1 organ dysfunction.
  ○ Septic shock (post–2016): vasopressor requirement to maintain mean arterial pressure ≥ 65 mmHg and lactate > 2 mmol/L despite adequate fluid resuscitation.
3. Receipt of at least one PMX-HP session during ICU admission.

#### 2.2.2. Exclusion Criteria

- Age < 20 years.
- Pregnancy.
- Known HIV infection or hemophilia.
- Solid-organ transplantation within 1 year.
- Cardiopulmonary resuscitation within 4 weeks prior to ICU admission.
- Anticipated survival < 30 days (e.g., terminal malignancy).
- Do-not-resuscitate (DNR) status at admission.
- End-stage liver failure (Child-Pugh class C).
- Allergy to polymyxin B, heparin, or extracorporeal circuits.
- Receipt of extracorporeal blood purification (CRRT, hemofiltration, or plasma exchange) within 24 h prior to PMX-HP initiation.

A total of 70 patients fulfilled initial screening criteria; 6 were excluded, leaving 64 patients in the final microbiological cohort.

### 2.3. Definitions of Infection Source and Microbiological Categories

Infection sources were classified based on clinical assessment, imaging, culture data, and attending physician documentation:

- Pneumonia
- Intra-abdominal infection (e.g., peritonitis, hepatobiliary infection, intra-abdominal abscess)
- Urinary tract infection (UTI)/urosepsis
- Skin and soft tissue infection (SSTI)
- Liver abscess
- Primary bacteremia
- Other/undetermined sources

Microbiological categories were defined as:

- Gram-negative bacteria (*E. coli*, *K. pneumoniae*, *A. baumannii*, *P. aeruginosa*, etc.)
- Gram-positive bacteria (*S. aureus*, *streptococci*, *enterococci*)
- Fungal pathogens (Candida, Aspergillus)
- No growth (negative cultures)
- Multidrug-resistant (MDR) as per international consensus: non-susceptibility to ≥1 agent in ≥3 antimicrobial categories.

Only pathogens documented in culture results from blood, respiratory specimens, sterile body fluids, or wound samples were included.

Pathogens were analyzed in aggregated categories (Gram-negative, Gram-positive, fungal, and no-growth) to ensure adequate statistical power and model stability. Subgroup analyses of individual organisms (e.g., *Escherichia coli* vs. *Acinetobacter baumannii*, *Candida* vs. *Aspergillus*) were not feasible due to limited case numbers within each pathogen subtype, which would have resulted in unstable estimates and overfitting in multivariable models. Therefore, pathogen aggregation was a pragmatic statistical decision commonly adopted in real-world sepsis cohort studies, and the results should be interpreted at the category level rather than as organism-specific effects.

### 2.4. PMX-HP Intervention

PMX-HP was administered using the Toraymyxin^®^ (PMX-20R) cartridge (Toray Industries, Tokyo, Japan). Each session typically lasted 2 h, with blood flow rates of 80–120 mL/min. Anticoagulation was performed with systemic unfractionated heparin per ICU protocol unless contraindicated.

The decision regarding the number of sessions, the timing of each PMX-HP session, and the use of sequential versus non-sequential strategies was at the discretion of the attending intensivist based on hemodynamic status, vasopressor requirement, infection severity, and overall prognosis.

For this study, we did not perform a comparison of PMX-HP strategies; instead, PMX-HP exposure was considered uniform, and the focus was placed on microbiological predictors.

### 2.5. Organ Support and Hemodynamic Parameters

The following ICU treatments were recorded:

- Continuous renal replacement therapy (CRRT)
  ○ Indications: severe AKI (KDIGO stage 3), anuria/oliguria, metabolic acidosis, or fluid overload.
  ○ Timing: early CRRT defined as initiation within 24 h of shock onset.
- Extracorporeal membrane oxygenation (ECMO)
  ○ Initiated for refractory hypoxemia or circulatory collapse.
- Vasoactive–inotropic score (VIS), calculated using the standard formula:

    VIS = dopamine + dobutamine + 100 × (epinephrine + norepinephrine) + 10   × milrinone + 10,000 × vasopressin VIS = dopamine + dobutamine + 100 ×  (epinephrine + norepinephrine) + 10 × milrinone + 10,000 × vasopressin VIS   = dopamine + dobutamine + 100 × (epinephrine + norepinephrine) + 10 ×       milrinone + 10,000 × vasopressin                                                                         

Vasoactive–inotropic score (VIS), calculated using the standard formula:

VIS = dopamine (μg/kg/min) + dobutamine (μg/kg/min)                              + 100 × epinephrine (μg/kg/min) + 100 × norepinephrine (μg/kg/min)     + 10 × milrinone (μg/kg/min) + 10,000 × vasopressin (U/min).       

VIS change (ΔVIS) was defined as T3–T1, with positive ΔVIS indicating worsening hemodynamic instability.

### 2.6. Microbiological and Infection-Source Variables

The following data were extracted:

- Pathogen species (culture-confirmed);
- MDR status;
- Infection source classification;
- Polymicrobial vs. monomicrobial infections;
- Culture-negative sepsis.

For statistical robustness, pathogen groups were analyzed in aggregated categories (Gram-negative, Gram-positive, fungal, no-growth). Subgroup evaluation for MDR organisms was performed prespecifically.

### 2.7. Outcomes

#### 2.7.1. Primary Outcome

- 28-day all-cause mortality.

#### 2.7.2. Secondary Outcomes

- ICU mortality;
- Hospital mortality;
- ICU length of stay (LOS);
- Hospital LOS;
- Requirement for CRRT (including early vs. late CRRT);
- Requirement for ECMO;
- Hemodynamic response (ΔVIS).

### 2.8. Statistical Analysis

Continuous variables were expressed as *mean ± standard deviation* (SD) or *median with interquartile range* (IQR) depending on distribution. Categorical variables were expressed as counts and percentages.

Comparisons were performed using:

- Student’s *t*-test (parametric continuous variables);
- Mann–Whitney U test (nonparametric continuous variables);
- Chi-square or Fisher’s exact test (categorical variables).

Variables with *p* < 0.10 in univariate analysis or considered clinically relevant (Gram-negative infection, MDR status, infection source, APACHE II, CRRT, VIS) were incorporated into multivariate logistic regression to identify independent predictors of 28-day mortality.

Adjusted odds ratios (OR) with 95% confidence intervals (CI) were reported.

All analyses were conducted using SPSS version 26 and R version 4.2. A two-tailed *p* < 0.05 was considered statistically significant.

### 2.9. Missing Data Handling

Pairwise deletion was used for missing variables. VIS time points were analyzed only if both T1 and T3 were available. Mortality outcomes had no missing data. No imputation was performed to avoid bias in microbiological subgroup analyses.

### 2.10. Ethical Approval

The study was approved by the Institutional Review Board of MacKay Memorial Hospital (IRB No. 18MMHIS198e). The waiver of informed consent was granted due to the retrospective de-identified nature of the study. All procedures adhered to the Declaration of Helsinki and institutional research policies.

## 3. Results

### 3.1. Baseline Characteristics

A total of 64 patients with severe sepsis or septic shock receiving PMX-HP were included. The mean age was 66.1 ± 12.3 years, and 67.2% were male. The median APACHE II score at ICU admission was 26 (IQR 21–32), indicating high severity of illness. Baseline demographics are summarized in Table 1.

### 3.2. Infection Sources and Pathogen Distribution

**Figure 1:**
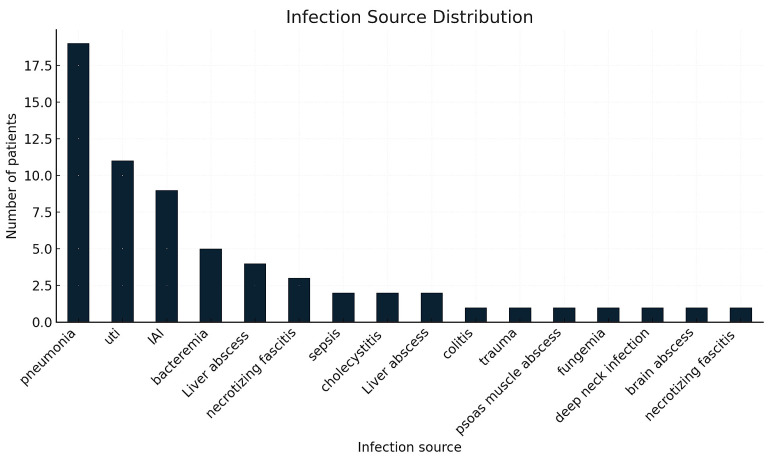
Distribution of primary infection sources among patients treated with PMX-HP. Legend: The bar chart illustrates the frequency of major infection sources in the cohort, including pneumonia, intra-abdominal infection, urinary tract infection, skin and soft-tissue infection, liver abscess, and other or undetermined origins. Values represent the number of patients per category.

The distribution of infection sources is presented in Table 2 and illustrated in Figure 1. Pneumonia was the most common source (29.7%), followed by intra-abdominal infection (18.8%), urinary tract infection (17.2%), skin/soft-tissue infection (10.9%), and liver abscess (9.4%). Other or indeterminate sources accounted for 14.1% of cases.

**Figure 2:**
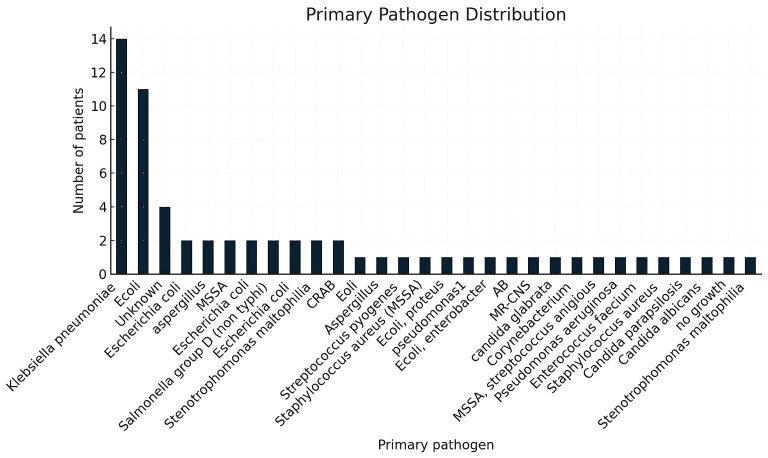
Distribution of primary pathogens identified at sepsis presentation. Legend: This figure shows the major pathogen categories isolated from clinical cultures, including Gram-negative bacteria, Gram-positive bacteria, fungal pathogens, less common organisms, and culture-negative cases. The chart displays the number of patients associated with each pathogen group.

Primary pathogen categories are also summarized in Table 2 and Figure 2. Gram-negative bacteria were predominant (70.3%), with *Escherichia coli* (31.3%) and *Klebsiella pneumoniae* (21.9%) being the most frequently identified organisms. Gram-positive organisms accounted for 12.5% of cases, fungal pathogens 7.8%, and 7.8% of patients had negative cultures.

**Figure 3:**
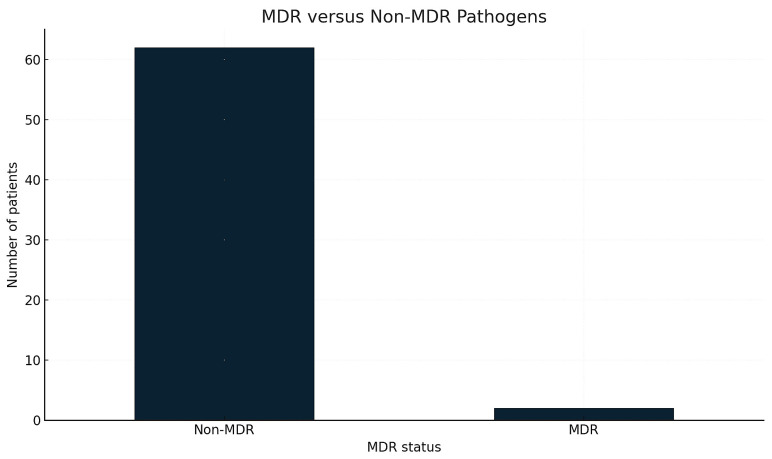
Comparison of multidrug-resistant (MDR) and non-MDR infections. Legend: The bar chart compares the number of patients with MDR pathogens versus non-MDR pathogens. MDR was classified based on routine microbiological reports indicating resistance to multiple antimicrobial classes. Values represent counts of patients in each resistance category.

Multidrug-resistant pathogens were identified in 26.6% of patients, with MDR distribution displayed in Figure 3.

### 3.3. Clinical Outcomes and Organ Support

Overall outcomes are summarized in Table 3. The 28-day mortality rate was 46.9%, ICU mortality 51.6%, and hospital mortality 53.1%. The median ICU length of stay was 9.3 days (IQR 4.4–21.1), and median hospital stay was 20.5 days (IQR 8.0–34.3).

Organ support requirements were considerable:

- CRRT within 24 h of shock onset: 45.3%
- CRRT at any time during the ICU stay: 67.2%
- ECMO support: 4.7%

### 3.4. Infection Source and Organ Dysfunction

Differences in organ support needs across infection sources are presented in Table 4. Intra-abdominal infection (91.7%) and “other/undetermined” infections (88.9%) had the highest CRRT utilization, whereas urinary tract infections required CRRT less frequently (18.2%).

However, 28-day mortality did not differ significantly among infection-source categories (*p* > 0.05). Liver abscess cases had a 0% mortality rate, whereas “other/undetermined” infections had the highest mortality (77.8%).

### 3.5. Pathogen Category, MDR Status, and Outcomes

As shown in Table 5, Gram-negative infections were not associated with higher 28-day mortality compared with non–Gram-negative or culture-negative cases (35.6% vs. 73.7%, *p* = NS). Similarly, MDR status was not significantly associated with mortality (52.9% vs. 43.2%, *p* = 0.42).

However, MDR infections were significantly associated with higher early CRRT requirement (64.7% vs. 38.6%, *p* = 0.048), indicating a greater burden of renal dysfunction.

### 3.6. Hemodynamic Response Based on VIS Change

VIS change within 24 h after PMX-HP was evaluated using norepinephrine dose differences. Patients were grouped into Increase (ΔVIS > 0) or No increase (ΔVIS ≤ 0) categories.

**Figure 4:**
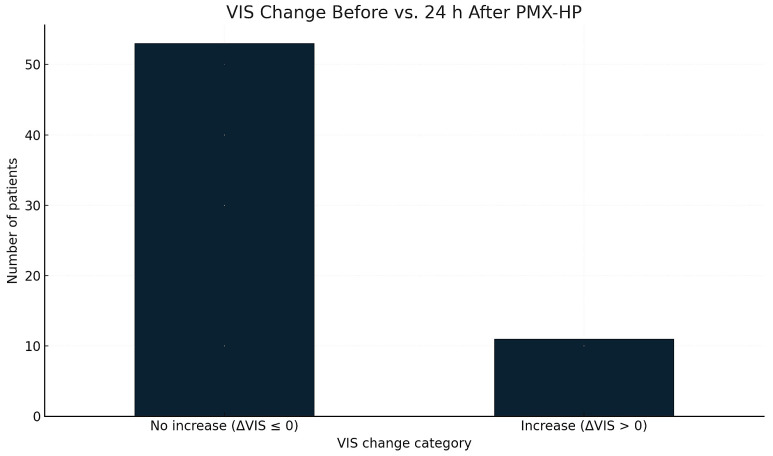
Changes in vasoactive-inotropic support following PMX-HP. Legend: The figure displays the number of patients who experienced an increase versus no increase in vasoactive-inotropic requirements within 24 h after PMX-HP. Patients were grouped according to whether their hemodynamic support needs worsened or remained stable/improved during this period.

Distribution of VIS categories is shown in Figure 4:

- Increase in VIS: 31.3%;
- No increase: 68.7%.

Patients with VIS increase had significantly higher mortality in univariate analysis (*p* < 0.01), indicating persistent hemodynamic instability.

### 3.7. Multivariate Predictors of 28-Day Mortality

A multivariate logistic regression model was used, including:

- APACHE II;
- CRRT (any time within 28 days);
- VIS change;
- Infection source;
- Pathogen category;
- MDR status.

Multivariate logistic regression analysis showed showed that only three factors independently predicted 28-day mortality:

1. APACHE II score (*p* = 0.02);
2. CRRT requirement (*p* = 0.01);
3. Positive VIS change (*p* = 0.006).

Microbiological variables—including infection source, Gram-negative status, and MDR status—were not independent predictors of mortality (Table 5).

## 4. Discussion

In this seven-year single-center cohort of adult patients with severe sepsis or septic shock receiving polymyxin B hemoperfusion, we found that microbiological category per se was not an independently associated factor of short-term mortality after adjustment for global severity and hemodynamic status. Instead, infection source, organ dysfunction burden, and vasopressor intensity at the time of hemoperfusion were the variables most strongly associated with outcome. These findings reinforce the concept that, in advanced sepsis, host response and shock physiology may dominate over the specific pathogen label when prognosis is considered [13,14,15,16].

APACHE II remains one of the most widely applied severity scores in the ICU and correlates closely with hospital mortality across a broad range of critical illnesses [13]. In our cohort, higher APACHE II at PMX-HP initiation was consistently associated with worse survival, which is in line with contemporary sepsis literature emphasizing that global physiologic derangement, rather than any single organ variable, captures the cumulative effect of infection, comorbidity, and treatment delay [14]. Sepsis is now understood as a life-threatening organ dysfunction caused by a dysregulated host response to infection, in which overlapping inflammatory, immune-paralysis, metabolic, and endothelial phenotypes coexist and evolve over time [14,15]. Large data-driven analyses have identified distinct clinical sepsis phenotypes with differing trajectories and treatment responsiveness [15], suggesting that therapies such as PMX-HP are likely to have heterogeneous effects depending on when and in whom they are applied. Our data, focused on patients already selected for PMX-HP, probably represent a subset with advanced, refractory phenotypes and substantial baseline risk, similar to more severe phenotypes described in previous work [15,16].

The high overall mortality in this study, despite aggressive bundle-based management, mirrors the persistent global burden of sepsis and the long-term vulnerability of survivors. Prior studies have highlighted that many patients remain at increased risk of death, rehospitalization, and functional decline for months to years after discharge, even when initial shock appears to have resolved [16]. Our findings therefore support the notion that PMX-HP is often deployed late, in a population where irreversible organ injury and immunologic exhaustion are already established, and consequently its incremental effect on “hard” outcomes is limited. Rather than contradicting previous reports, our results fit into a broader picture in which timing, patient phenotype, and concomitant supportive care critically modulate the apparent effect size of any adjunctive therapy [14,15,16].

A key novel observation is the strong prognostic contribution of vasopressor intensity—summarized by the vasoactive–inotropic score (VIS)—at the time of hemoperfusion. VIS has been validated as an early, dynamic marker of circulatory failure and mortality in adult sepsis cohorts [17]. In our analysis, higher VIS was independently associated with mortality even after controlling for APACHE II and comorbidities, underscoring that the degree of catecholamine dependence at PMX-HP initiation captures a dimension of cardiovascular and microcirculatory collapse that is not fully reflected by static severity scores. This is concordant with emerging hemodynamic-phenotype literature, which suggests that patients with profound vasoplegia and high vasopressor requirements represent a particularly high-risk group in whom the window for reversing shock is narrow [17,18]. Peripheral perfusion–targeted resuscitation strategies, such as those evaluated in the ANDROMEDA-SHOCK trial, have shown that rapid normalization of simple bedside markers like capillary refill time is associated with less organ dysfunction and a trend toward lower mortality compared with lactate-guided strategies [18]. In our cohort, however, PMX-HP was often initiated at a stage where VIS remained high despite aggressive resuscitation, implying that the opportunity to leverage microcirculatory improvement may already have been missed.

The 24 h time point for VIS reassessment was selected to capture the early hemodynamic trajectory following PMX-HP initiation, while minimizing confounding from later-stage clinical deterioration, rescue therapies, or withdrawal of care. Prior sepsis studies have demonstrated that changes in vasoactive requirements within the first 24 h are strongly associated with outcomes and reflect the balance between shock resolution and persistent vasoplegia. Accordingly, ΔVIS within 24 h was used as a pragmatic dynamic indicator of early circulatory response in this cohort. Although alternative time points may provide complementary information, the present approach is consistent with prior literature emphasizing early hemodynamic trends as prognostic markers in septic shock.

Our data should also be interpreted in the context of other adjunctive therapies for septic shock. Large randomized trials of systemic corticosteroids, such as ADRENAL and APROCCHSS, demonstrated faster shock reversal and, in one trial, a modest reduction in mortality, but the overall survival benefit remains debated and appears to depend on baseline risk and co-interventions [19,20]. More recently, combination “metabolic resuscitation” protocols that incorporate vitamin C, thiamine, and hydrocortisone have been tested in multiple randomized trials. CITRIS-ALI reported no significant improvement in organ dysfunction scores or inflammatory biomarkers with high-dose vitamin C compared with placebo [21], and both the VITAMINS and VICTAS trials failed to show an advantage of vitamin C–thiamine–steroid combinations over standard care in terms of vasopressor-free days or mortality [22,23]. The LOVIT trial even raised safety concerns, with high-dose vitamin C associated with higher rates of death or persistent organ dysfunction [24]. Taken together, these data illustrate that even biologically plausible adjuncts, when evaluated in rigorously conducted trials, often show neutral or context-dependent effects. PMX-HP should be viewed within this same framework: promising mechanistic rationale and early uncontrolled reports, contrasted against mixed signals in contemporary randomized and observational studies [19,20,21,22,23,24].

Multiple systematic reviews and meta-analyses have synthesized the evidence for polymyxin B hemoperfusion in sepsis and septic shock. Early pooled analyses suggested potential survival benefits in selected populations, particularly in abdominal sepsis with high endotoxin burden [25,26]. However, later meta-analyses incorporating more recent randomized trials and propensity-matched cohorts have reported attenuated or absent mortality benefit, with substantial between-study heterogeneity and risk of bias [26,27,28]. Some analyses suggest that any advantage might be confined to patients with intermediate severity or clearly elevated endotoxin activity, whereas neutral or harmful effects cannot be excluded in other subgroups [25,26,27,28]. Case–control and small prospective studies combining PMX-HP with continuous renal replacement therapy have shown more rapid endotoxin or inflammatory mediator clearance but no consistent improvement in clinical endpoints [29,30]. Our findings are concordant with this evolving literature: in a real-world cohort where PMX-HP was integrated into complex sepsis care pathways, mortality remained high and appeared to be driven mainly by global severity, infection source, and vasopressor burden, rather than by microbiological category alone.

The strong signal from infection source in our multivariable models is clinically intuitive. Abdominal and pulmonary foci are consistently associated with higher mortality and greater organ dysfunction in sepsis epidemiology studies, reflecting both the difficulty of achieving timely, effective source control and the potential for ongoing contamination or hypoxic injury [14]. In our cohort, patients with abdominal or respiratory sources not only had higher baseline APACHE II scores but also required more intensive vasopressor support, reinforcing the importance of early, aggressive source control and individualized hemodynamic targets in these populations. Beyond the categorical label, infection source also interacts with coagulation and endothelial injury pathways; for example, intra-abdominal infection is often associated with profound endotoxemia and microvascular thrombosis, which may partially explain why PMX-HP was originally hypothesized to be most effective in this subgroup [25,26,27,30].

Coagulation abnormalities are another key dimension of septic shock pathophysiology that may influence both prognosis and response to extracorporeal therapies. Sepsis-induced coagulopathy (SIC) and overt disseminated intravascular coagulation have been associated with higher mortality and may represent potential therapeutic targets [31,32]. The SIC score, incorporating platelet count, prothrombin time, and organ dysfunction, has been validated as a simple tool to identify high-risk patients and to enrich clinical trials of anticoagulant therapies [31]. Recent reviews emphasize that SIC is a dynamic continuum rather than a binary event, evolving from compensated pro-thrombotic changes to decompensated consumption coagulopathy, and that the timing of intervention is crucial [32]. Although our dataset did not include full SIC components for all patients, the high rates of hepatic and renal dysfunction, together with elevated VIS, suggest that many of our patients were in advanced stages of immunothrombosis. In such a context, PMX-HP aimed at endotoxin removal may be insufficient to reverse the downstream coagulopathic cascade once microvascular damage is established.

From an immunologic perspective, modern models of sepsis describe a complex interplay of simultaneous hyper-inflammation, immune paralysis, endothelial disruption, and metabolic rewiring [14,33]. Comprehensive reviews of sepsis immunology highlight that circulating endotoxin represents only one component within a broader pattern of pathogen- and damage-associated molecular patterns and dysregulated cytokine networks [14,33]. It is therefore unsurprising that targeting a single upstream mediator such as endotoxin with PMX-HP has not consistently translated into survival benefit, particularly when applied late in the disease course. Our observation that infection source and vasopressor dependence outperform microbiological category in predicting outcome reinforces the idea that the overall immune-hemodynamic phenotype is more informative for prognosis and treatment selection than the organism label alone [15,17,18,33].

The real-world nature of our cohort also underscores the challenge of translating trial results into routine practice. Sepsis incidence estimates derived from clinical criteria show persistently high mortality and resource use, emphasizing that a large fraction of ICU capacity continues to be devoted to these patients [34]. Our single-center Taiwanese experience with PMX-HP aligns with other recent real-world reports from Asian ICUs and registry data suggesting that patients selected for hemoperfusion frequently have extreme severity, multidrug-resistant infection, and a high burden of organ support [26,30,35]. Differences in case-mix, timing of referral, availability of surgical and interventional radiology services, and local antimicrobial resistance patterns likely contribute to the variability in observed outcomes across centers and regions.

Several limitations merit consideration. First, the retrospective single-center design precludes causal inference regarding PMX-HP effectiveness; unmeasured confounding and treatment-by-indication bias are unavoidable. Second, we lacked standardized endotoxin activity assays and serial immunologic markers, limiting our ability to explore mechanistic responders versus non-responders. Third, our sample size, although reflecting all eligible PMX-HP cases over seven years, may still be underpowered for detecting modest interactions between microbiological category and hemodynamic or coagulation phenotypes. Finally, we did not compare PMX-HP with alternative extracorporeal strategies such as cytokine adsorbers or high-cutoff membranes, which are emerging as potential options in selected severe sepsis phenotypes [30].

Despite these limitations, our findings have practical implications. For clinicians, they suggest that in patients considered for PMX-HP, attention should focus on global severity (APACHE II), vasopressor intensity (VIS), and infection source rather than on microbiological category alone when counseling families and allocating resources. For investigators, our data support the design of future trials that stratify by hemodynamic and coagulation phenotypes, incorporate robust endotoxin and immune-function measurements, and test PMX-HP earlier in the disease trajectory, before irreversible organ failure is established. Finally, as national and international sepsis registries expand, integration of granular real-world PMX-HP data—similar to ours and other recent cohorts—will be essential to clarify which patients, if any, derive a clinically meaningful survival or recovery benefit from this demanding and costly therapy [25,26,27,28,29,30,34,35].

### Implications for Nursing Practice

In ICUs where polymyxin B hemoperfusion (PMX-HP) is implemented, bedside nurses play a central role in early recognition of circulatory deterioration, organ dysfunction surveillance, and safe extracorporeal therapy delivery. Our findings suggest that short-term prognosis in this PMX-HP-treated cohort is driven primarily by global illness severity, renal failure requiring CRRT, and persistent vasopressor dependence (VIS dynamics), rather than by microbiological category alone.

Accordingly, nursing priorities may include rigorous, time-stamped documentation of vasoactive agents to enable reliable VIS trending before and after PMX-HP; proactive surveillance for acute kidney injury, urine output, and fluid balance to facilitate timely CRRT escalation; coordination of diagnostic and source-control pathways (e.g., imaging and drainage procedures) when pneumonia or intra-abdominal sources are suspected; and standardized safety checks during PMX-HP/CRRT, including vascular access assessment, anticoagulation monitoring per protocol, circuit pressure alarms, and continuous hemodynamic monitoring.

Embedding these elements into a structured nursing checklist may support earlier escalation of care and more consistent multidisciplinary communication in refractory septic shock.

## 5. Conclusions

In this real-world cohort of adults with severe sepsis and septic shock treated with polymyxin B hemoperfusion, we observed substantial hemodynamic improvement—reflected by reductions in vasopressor requirements—particularly among patients with severe vasodilatory shock. However, microbiological variables, including pathogen category, infection source, and multidrug-resistant status, were not independent predictors of 28-day mortality. Instead, severity of illness, the need for renal replacement therapy, and persistent vasopressor dependence were the principal determinants of outcome.

These findings suggest that polymyxin B hemoperfusion should be viewed as an adjunct to, rather than a replacement for, optimized antimicrobial therapy, prompt source control, and high-quality critical care. Future multicenter studies should incorporate precise patient selection based on hemodynamic and coagulation profiles, as well as objective measures of endotoxin activity, to identify those most likely to benefit. Until such data are available, the decision to initiate PMX-HP should be individualized, balancing potential hemodynamic gains against resource consumption and the current uncertainty regarding survival benefit.

## Figures and Tables

**Table 1:** Baseline characteristics of the study cohort (n = 64). Legend: This table summarizes the demographic and clinical characteristics of patients receiving PMX-HP, including age, sex distribution, body weight, and severity of illness measured by APACHE II.

Variable	Value
Age, years, mean ± SD	66.1 ± 12.3
Male sex, *n* (%)	43 (67.2%)
Body weight, kg, mean ± SD	66.5 ± 15.2
APACHE II score, median (IQR)	26 (21–32)

**Table 2:** Infection Sources and Pathogen Categories. Legend: This table presents the distribution of primary infection sources and major pathogen categories, including Gram-negative, Gram-positive, fungal, and culture-negative sepsis.

A. Infection Source Distribution	
Infection source category	*n* (%)
Pneumonia	19 (29.7%)
Intra-abdominal infection	12 (18.8%)
Urinary tract infection	11 (17.2%)
Skin/soft tissue infection	7 (10.9%)
Liver abscess	6 (9.4%)
Other/undetermined	9 (14.1%)
Total	64 (100%)
**B. Pathogen Category Distribution**	
Pathogen category	*n* (%)
Gram-negative bacteria	45 (70.3%)
Gram-positive bacteria	8 (12.5%)
Fungal pathogens	5 (7.8%)
Other bacteria	1 (1.6%)
No growth/unknown	5 (7.8%)
Total	64 (100%)

**Table 3:** Overall outcomes and organ support. Legend: This table shows major clinical outcomes, including ICU and hospital mortality, length of stay, and requirements for CRRT and ECMO during admission.

Outcome/Organ Support	Value
ICU length of stay, median (IQR)	9.3 (4.4–21.1)
Hospital length of stay, median (IQR)	20.5 (8.0–34.3)
28-day mortality	30 (46.9%)
ICU mortality	33 (51.6%)
Hospital mortality	34 (53.1%)
CRRT within 24 h	29 (45.3%)
CRRT within 28 days	43 (67.2%)
ECMO use	3 (4.7%)

**Table 4:** Infection source versus CRRT requirement and 28-day mortality. Legend: This table compares organ support needs and mortality across different infection-source groups, illustrating how source of infection correlates with renal failure and clinical outcomes.

Infection Source	*n*	CRRT Any Time	28-Day Mortality
Pneumonia	19	14 (73.7%)	12 (63.2%)
Intra-abdominal infection	12	11 (91.7%)	6 (50.0%)
Urinary tract infection	11	2 (18.2%)	1 (9.1%)
Skin/soft tissue infection	7	4 (57.1%)	4 (57.1%)
Liver abscess	6	4 (66.7%)	0 (0.0%)
Other/undetermined	9	8 (88.9%)	7 (77.8%)
Total	64	43 (67.2%)	30 (46.9%)

**Table 5:** Pathogen category versus CRRT requirement and mortality. Legend: This table evaluates the association between pathogen category and clinical severity, including mortality and the need for renal replacement therapy.

Pathogen Category	*n*	28-Day Mortality	CRRT Any Time
Gram-negative bacteria	45	16 (35.6%)	28 (62.2%)
Non–Gram-negative/others	19	14 (73.7%)	15 (78.9%)
Total	64	30 (46.9%)	43 (67.2%)

## Data Availability

The datasets generated and analyzed during the current study are available from the corresponding author on reasonable request. All relevant clinical data are presented within the article.

## References

[1] Rudd K.E., Johnson S.C., Agesa K.M., Shackelford K.A., Tsoi D., Kievlan D.R., Colombara D.V., Ikuta K.S., Kissoon N., Finfer S. (2020). Global, regional, and national sepsis incidence and mortality, 1990-2017: Analysis for the Global Burden of Disease Study. Lancet.

[2] Evans L., Rhodes A., Alhazzani W., Antonelli M., Coopersmith C.M., French C., Machado F.R., McIntyre L., Ostermann M., Prescott H.C. (2021). Surviving Sepsis Campaign: International guidelines for management of sepsis and septic shock 2021. Intensive Care Med..

[3] Singer M., Deutschman C.S., Seymour C.W., Shankar-Hari M., Annane D., Bauer M., Bellomo R., Bernard G.R., Chiche J.D., Coopersmith C.M. (2016). The Third International Consensus Definitions for Sepsis and Septic Shock (Sepsis-3). JAMA.

[4] Hotchkiss R.S., Moldawer L.L., Opal S.M., Reinhart K., Turnbull I.R., Vincent J.L. (2016). Sepsis and septic shock. Nat. Rev. Dis. Primers.

[5] Shumba P., Mairpady Shambat S., Siemens N. (2019). The Role of Streptococcal and Staphylococcal Exotoxins and Proteases in Human Necrotizing Soft Tissue Infections. Toxins.

[6] Song J., Cho H., Park D.W., Moon S., Kim J.Y., Ahn S., Lee S.-G., Park J. (2021). Vasoactive-inotropic score as an early predictor of mortality in adult patients with sepsis. J. Clin. Med..

[7] Meyhoff T.S., Hjortrup P.B., Moller M.H., Haase N., Perner A., Wetterslev J., Sivapalan P., Laake J.H., Cronhjort M., Jakob S.M. (2022). Restriction of intravenous fluid in ICU patients with septic shock (CLASSIC trial). N. Engl. J. Med..

[8] Russell J.A., Walley K.R., Singer J., Gordon A.C., Hebert P.C., Cooper D.J., Holmes C.L., Mehta S., Granton J.T., Storms M.M. (2008). Vasopressin versus norepinephrine infusion in patients with septic shock. N. Engl. J. Med..

[9] Seymour C.W., Kennedy J.N., Wang S., Chang C.-C.H., Elliott C.F., Xu Z., Berry S., Clermont G., Cooper G., Gomez H. (2019). Derivation, validation, and potential treatment implications of novel clinical phenotypes of sepsis. JAMA.

[10] Vincent J.L., Opal S.M., Marshall J.C., Tracey K.J. (2013). Sepsis definitions: Time for change. Lancet.

[11] Cecconi M., Evans L., Levy M., Rhodes A. (2018). Sepsis and septic shock. Lancet.

[12] Schmidt M., Pham T., Arcadipane A., Hajage D., Lebreton G., Monsel A., Voiriot G., Levy D., Muller G., Hekimian G. (2020). Extracorporeal membrane oxygenation for severe acute respiratory distress syndrome associated with COVID-19: A retrospective cohort study. Lancet Respir. Med..

[13] Brechot N., Luyt C.-E., Schmidt M., Leprince P., Trouillet J.-L., Leger P., Pavie A., Chastre J., Legrand M., Combes A. (2013). Venoarterial extracorporeal membrane oxygenation support for refractory cardiovascular dysfunction during severe bacterial septic shock. Crit. Care Med..

[14] Aubron C., Cheng A.C., Pilcher D., Leong T., Magrin G., Cooper D.J. (2013). Factors associated with outcomes of patients on extracorporeal membrane oxygenation support: A 5-year cohort study. Crit. Care.

[15] Schlapbach L.J., Schlapbach H.R., Biclard K., Danton M., Poryo M., Agha M., Henderson N., Broman L.M., MacLaren G. (2019). Defining benefit threshold for extracorporeal membrane oxygenation in children with sepsis-a binational multicenter cohort study. Crit. Care.

[16] Chen Y.-C., Chang S.-C., Pu C., Tang G.-J. (2013). The impact of nationwide education program on clinical practice in sepsis care and mortality of severe sepsis: A population-based study in Taiwan. PLoS ONE.

[17] Chang W.-H., Yang S.H., Shen H.-F., Hu T.-Y., Wu W.-J. (2025). Impact of continuous renal replacement therapy on outcomes in septic shock patients receiving polymyxin B hemoperfusion: A retrospective cohort study. Biomedicines.

[18] Nishida O., Ogura H., Egi M., Fujimi S., Jinzaki M., Nakamura M., Iba T., Iriyama H., Ito N., Uchida M. (2021). The Japanese clinical practice guidelines for management of sepsis and septic shock 2020. J. Intensive Care.

[19] Cutuli S.L., Artigas A., Fumagalli R., Monti G., Ranieri V.M., Ronco C., Antonelli M. (2016). The EUPHAS 2 Collaborative Group. Polymyxin-B hemoperfusion in septic patients: Analysis of a multicenter registry. Ann. Intensive Care.

[20] Chang W.-H., Hu T.-Y., Kuo L.-K. (2025). Real-world outcomes and prognostic factors of polymyxin B hemoperfusion in severe sepsis and septic shock: A seven-year single-center cohort study from Taiwan. Life.

[21] Chang W.-H., Hu T.-Y., Kuo L.-K. (2026). Sequential Versus Non-Sequential Polymyxin B Hemoperfusion in Severe Sepsis and Septic Shock: A Real-World Cohort Analysis of Survival in an Asian ICU. Diagnostics.

[22] Dellinger R.P., Bagshaw S.M., Antonelli M., Foster D.M., Klein D.J., Marshall J.C., Palevsky P.M., Weisberg L.S., Schorr C.A., Trzeciak S. (2018). Effect of targeted polymyxin B hemoperfusion on 28-day mortality in patients with septic shock and elevated endotoxin level: The EUPHRATES randomized clinical trial. JAMA.

[23] Payen D., Guilhot J., Launey Y., Lukaszewicz A.-C., Joannes-Boyau O., Joffre J., Mira J.-P., Perny A., Rousseau C., Reignier J. (2015). Early use of polymyxin B hemoperfusion in abdominal septic shock: The ABDOMIX randomized clinical trial. Intensive Care Med..

[24] Cruz D.N., Antonelli M., Fumagalli R., Foltran F., Brienza N., Donati A., Malcangi V., Petrini F., Volta G., Ronco C. (2009). Early use of polymyxin B hemoperfusion in abdominal septic shock: The EUPHAS randomized controlled trial. JAMA.

[25] Shoji H. (2021). Therapeutic rationale for endotoxin removal with polymyxin B immobilized fiber column (Toraymyxin). Int. J. Mol. Sci..

[26] Li X., Liu C., Mao Z., Qi S., Song R., Zhou F. (2021). Effectiveness of polymyxin B-immobilized hemoperfusion against sepsis and septic shock: A systematic review and meta-analysis. J. Crit. Care.

[27] Chen J.J., Lai P.C., Lee T.H., Huang Y.T. (2023). Blood purification for adult patients with severe infection or sepsis: A network meta-analysis of randomized controlled trials. Crit. Care Med..

[28] Chen N., Zhou M., Dong X., Qu J., Gong F., Han Y., Qiu Y., Wang J., Liu Y., Wei Y. (2020). Epidemiological and clinical characteristics of 99 cases of 2019 novel coronavirus pneumonia in Wuhan, China: A descriptive study. Lancet.

[29] Wiersinga W.J., Rhodes A., Cheng A.C., Peacock S.J., Prescott H.C. (2020). Pathophysiology, transmission, diagnosis, and treatment of coronavirus disease 2019 (COVID-19): A review. JAMA.

[30] Angus D.C., van der Poll T. (2013). Severe sepsis and septic shock. N. Engl. J. Med..

[31] Broman L.M., Dubrovskaja O., Balik M. (2023). Extracorporeal membrane oxygenation for septic shock in adults and children: A narrative review. J. Clin. Med..

[32] Aubron C., Cheng A.C., Pilcher D., Pellegrino V., Davies A., Cooper D.J. (2013). Infections acquired by adults who receive extracorporeal membrane oxygenation: Risk factors and outcome. Infect. Control Hosp. Epidemiol..

[33] Beretta-Piccoli X., Biarent D., De Bels D., Honore P.M., Redant S. (2020). ECMO in paediatric septic shock: An urgent need for prospective trial. Crit. Care.

[34] Reinhart K., Daniels R., Kissoon N., Machado F.R., Schachter R.D., Finfer S. (2017). Recognizing sepsis as a global health priority-A WHO resolution. N. Engl. J. Med..

[35] Deinhardt-Emmer S., Chousterman B.G., Schefold J.C., Flohe S.B., Skirecki T., Kox M., Winkler M.S., Cossarizza A., Wiersinga W.J., van der Poll T. (2025). Sepsis in patients who are immunocompromised: Diagnostic challenges and future therapies. Lancet Respir. Med..

